# Structures of dicobalt and dinickel 4,4′-bi­phenyldi­carboxyl­ate di­hydroxide, *M*_2_(O_2_CC_6_H_4_C_6_H_4_CO_2_)(OH)_2_, *M* = Co and Ni, and di­ammonium 4,4′-bi­phenyldi­carboxyl­ate from powder diffraction data

**DOI:** 10.1107/S2056989022009288

**Published:** 2022-09-30

**Authors:** Joshua D. Vegetabile, James A. Kaduk

**Affiliations:** a Department of Chemistry, North Central College, 131 S. Loomis St., Naperville IL 60540, USA; University of Aberdeen, Scotland

**Keywords:** powder diffraction, density functional theory, bi­phenyldi­carboxyl­ate, cobalt, nickel, manganese, hydroxide, ammonium

## Abstract

The triclinic structures of poly[(μ_4_-4,4′-bi­phenyldi­carboxyl­ato)di-μ-hydroxido-dicobalt], [Co_2_(C_14_H_8_O_4_)(OH)_2_]_*n*_, and poly[(μ_4_-4,4′-bi­phenyldi­carboxyl­ato)di-μ-hydroxido-dinickel], [Ni_2_(C_14_H_8_O_4_)(OH)_2_]_*n*_, were established using laboratory X-ray powder diffraction data. These structures, as well as that of poly[(μ_4_-4,4′-bi­phenyldi­carboxyl­ato)di-μ-hydroxido-dimanganese], [Mn_2_(C_14_H_8_O_4_)(OH)_2_]_*n*_, were optimized using density functional techniques. The structure of di­ammonium 4,4′-bi­phenyldi­carboxyl­ate, 2NH_4_^+^·C_14_H_8_O_4_^2−^, was also solved using laboratory powder data. The Mn and Co compounds are isostructural: the octa­hedral MO_6_ groups share edges to form chains running parallel to the *c*-axis. These chains share corners (OH groups) to link into layers lying parallel to the *bc* plane. The hydroxyl groups do not participate in hydrogen bonds. The structure of (NH_4_)_2_BPDC consists of alternating layers of BPDC and ammonium ions lying parallel to the *ab* plane. Each hydrogen atom of the ammonium ions in (NH_4_)_2_BPDC participates in a strong N—H⋯O hydrogen bond.

## Abstract 2

The triclinic structures of dicobalt and dinickel 4,4′-bi­phenyldi­carboxyl­ate di­hydroxide, *M*_2_(O_2_CC_6_H_4_C_6_H_4_CO_2_)(OH)_2_ were established using laboratory X-ray powder diffraction data. These structures, as well as that of Mn_2_(O_2_CC_6_H_4_C_6_H_4_CO_2_)(OH)_2_, were optimized using density functional techniques. The structure of di­ammonium 4,4′-bi­phenyldi­carboxyl­ate was also solved using laboratory powder data.

## 1. Chemical context

Metal–organic frameworks (MOFs) are a class of compounds that have both organic (linker mol­ecule) and inorganic (metal node) components. MOFs are used in many applied areas of science, such as gas separation and catalysis, but often the crystal structures of these MOFs are not reported. Knowing the crystal structures of MOFs lets us understand them at a mol­ecular level as well as identify them more efficiently.

**Figure 1:**
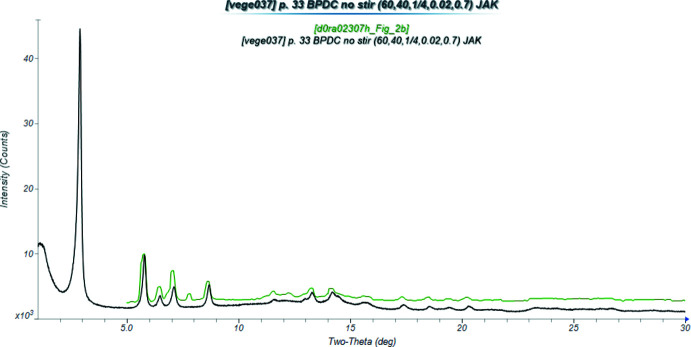
Comparison of the powder pattern of the Co_2_BPDC(OH)_2_ of this study (black) to that reported by Ipadeola & Ozoemena (2020 ▸; green). The literature pattern (measured using Cu *K*α radiation) was digitized using *UN-SCAN-IT* (Silk Scientific, 2013 ▸), and converted to Mo *K*α using *JADE Pro* (MDI, 2021 ▸). Image generated using *JADE Pro* (MDI, 2021 ▸).

From an attempt to prepare a porous Co-BPDC (BPDC = 4,4′-bi­phenyldi­carboxyl­ate, C_14_H_8_O_4_^2–^) MOF we obtained a dense Co-BPDC phase previously synthesized by Ipadeola & Ozoemena (2020 ▸). They reported a powder pattern, but did not otherwise characterize the compound, as it was decomposed to make nano-Co_3_O_4_. Their XRD pattern was similar to ours, but they did not measure to a low-enough angle to observe the strongest peak of the pattern (Fig. 1 ▸).

**Figure 2:**
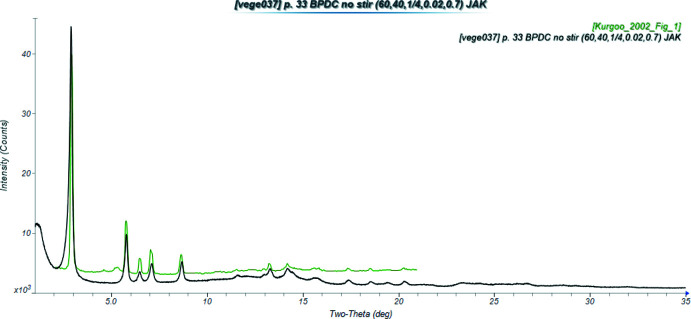
Comparison of the powder pattern of the Co_2_BPDC(OH)_2_ of this study (black) to that reported by Kurmoo & Kumagai (2002 ▸; green). The literature pattern (measured using Cu *K*α radiation) was digitized using *UN-SCAN-IT* (Silk Scientific, 2013 ▸), and converted to Mo *K*α using *JADE Pro* (MDI, 2021 ▸). Image generated using *JADE Pro* (MDI, 2021 ▸).

The magnetic properties of Co_2_BPDC(OH)_2_ were studied by Kurmoo & Kumagai (2002 ▸) and an X-ray powder pattern was provided (Fig. 2 ▸). They stated that the compound was isostructural to the analogous terephthalate. That structure was reported to crystallize in space group C2/*m*, which we believe to be incorrect (Markun *et al.*, 2022 ▸).

Most syntheses involving BPDC use H_2_BPDC and a base. We prepared di­ammonium 4,4-bi­phenyldi­carboxyl­ate as an alternative (and more soluble) reagent, characterized its crystal structure, and used it to prepare Ni_2_BPDC(OH)_2._ 
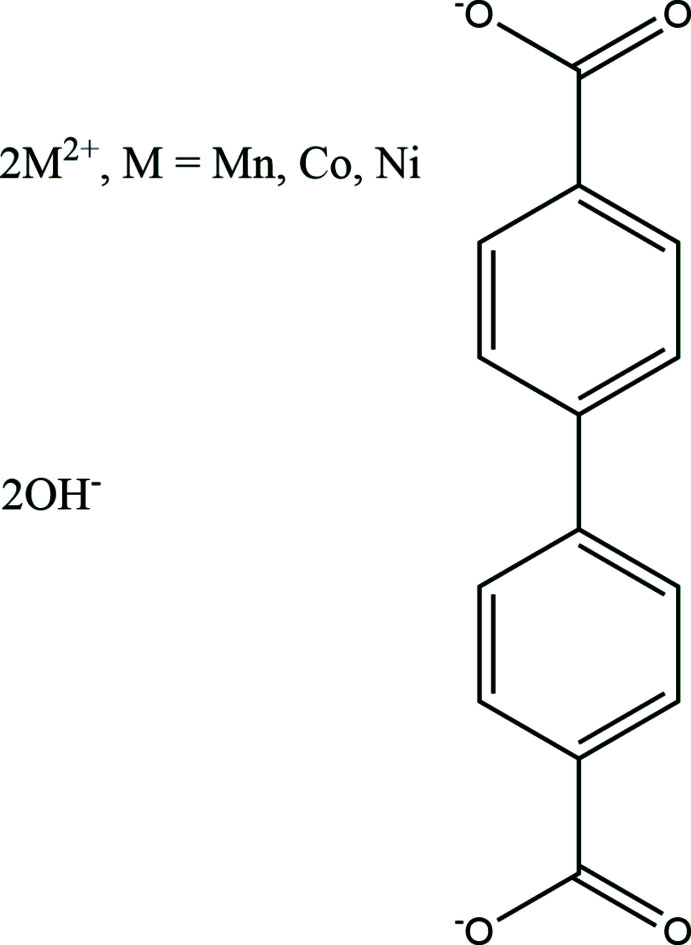
 
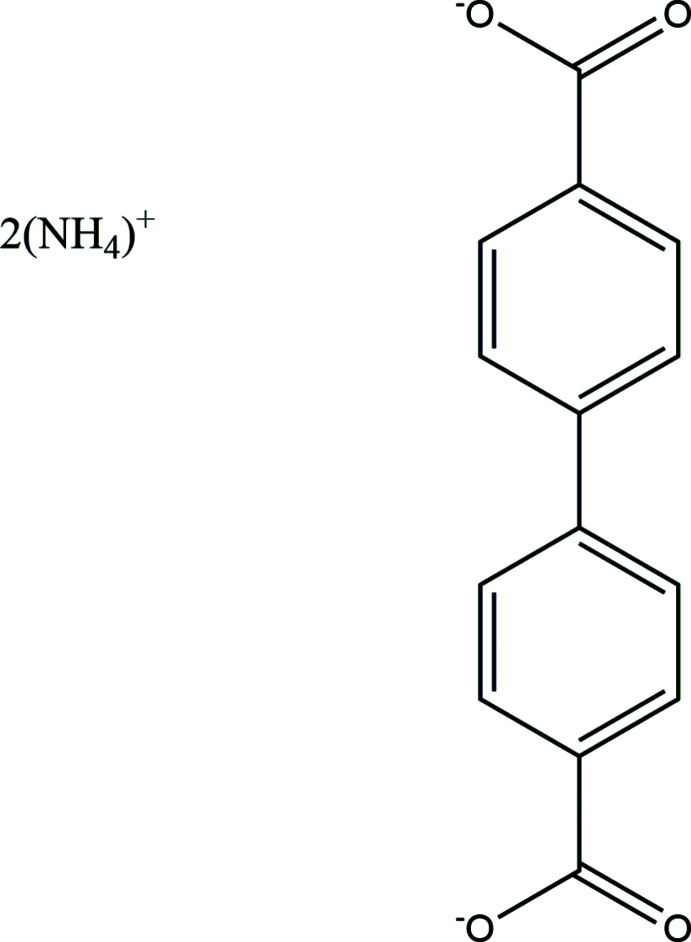


## 2. Structural commentary

**Figure 3:**
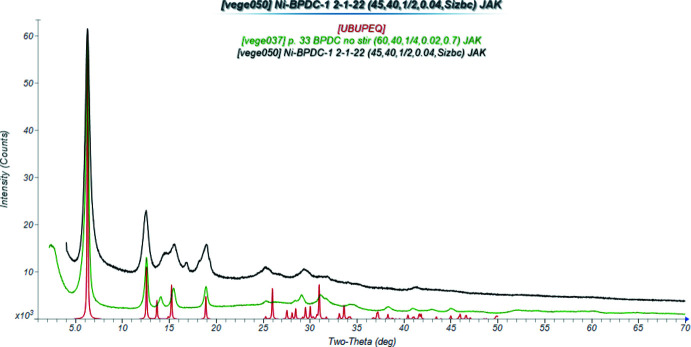
Powder patterns of Mn_2_BPDC(OH)_2_ (calculated from CSD entry UBUPEQ; red) to the experimental patterns of Co2BPDC(OH)2 (green) and Ni_2_BPDC(OH)_2_ (black). The patterns were converted to Cu *K*α using *JADE Pro* (MDI, 2021 ▸). Image generated using *JADE Pro* (MDI, 2021 ▸).

**Figure 4:**
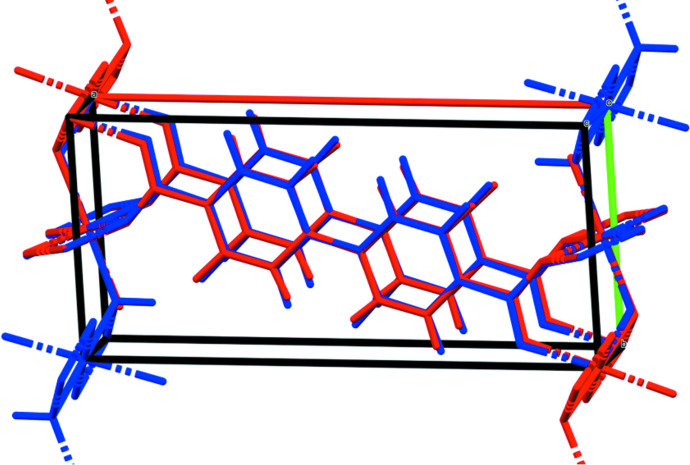
Comparison of the Rietveld-refined (red) and *VASP*-optimized (blue) structures of Mn_2_(BPDC)(OH)_2_. The r.m.s. Cartesian displacement is 0.133 Å. Image generated using *Mercury* (Macrae *et al.*, 2020 ▸).

The X-ray powder patterns show that the *M*_2_BPDC(OH)_2_ phases for *M* = Mn, Co, and Ni are isostructural (Fig. 3 ▸). The root-mean-square Cartesian displacements between the experimental (single crystal or Rietveld-refined) and DFT-optimized structures are 0.133, 0.264, and 0.563 Å for *M* = Mn, Co, and Ni, respectively (Figs. 4 ▸–6 ▸ ▸). The value for nickel is outside of the normal range for correct structures (van de Streek & Neumann, 2014 ▸). The behavior of the structure during various refinements and optimizations suggests that there might be alternate orientations of the BPDC ligand and alternate coordination of the Ni cations. Sorting out these details is not supported by the relatively poor diffraction data on the Ni compound. This discussion concentrates on the DFT-optimized structures.

All of the bond distances, angles, and torsion angles in the BPDC anions fall within the normal ranges indicated by a *Mercury* Mogul Geometry check (Macrae *et al.*, 2020 ▸). The O12—C11—C5—C6 torsion angles (which represent the twist of the carboxyl­ate group out of the phenyl ring plane) of −13.1, −14.1, and −6.6° for Mn, Co, and Ni, respectively, represent increases of conformational energy of approximately 1 kcal mol^−1^ (Kaduk *et al.*, 1999 ▸). These small increases can be easily overcome by energy gains in coordination to the metal ions. The C8—C10—C10—C1 torsion angles of 0.6, 0.6, and 0.1° indicate that the BPDC ligands are essentially planar. The approximate Miller planes of the benzene rings of the BPDC moieties are (238), (225) and (259) for Mn, Co, and Ni, respectively.

**Figure 7:**
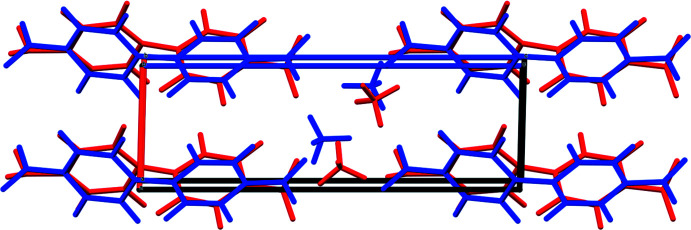
Comparison of the Rietveld-refined (red) and *VASP*-optimized (blue) structures of (NH_4_)_2_(BPDC). The r.m.s. Cartesian displacement is 0.384 Å. Image generated using Mercury (Macrae *et al.*, 2020 ▸).

Unlike the metal complexes, in di­ammonium BPDC, the aromatic rings are nearly perpendicular (C2—C4—C11—C14 = 85.7°). One carboxyl­ate group lies nearly in the ring plane (O25—C21—C12—C15 = 4.6°), while the other (O24—C22—C6—C3 = 85.6°) is nearly perpendicular to its ring. The r.m.s. Cartesian displacement of the non-H atoms in the BPDC anion is 0.384 Å (Fig. 7 ▸).

Analysis of the contributions to the total crystal energy of the structures using the Forcite module of *Materials Studio* (Dassault Systèmes, 2021 ▸) suggests that bond and angle distortion terms dominate the intra­molecular deformation energy in all three metal compounds. The inter­molecular energy in all three compounds is dominated by electrostatic attractions, which represent the *M*—O coordinate bonds.

The density of states (DOS) calculated by *VASP* (Kresse & Furthmüller, 1996 ▸) indicate that all three *M*-BPDC compounds are semiconductors, with band gaps of 1.695, 1.407 and 0.856 eV for Mn, Co and Ni respectively. Both the HOMO and LUMO consist mainly of metal *d* states. For Mn and Co, the DOS for the up and down spins differ, while for Ni they are very similar. Thus, the bonding in the Ni compound seems to be different than that in the other two.

A uniaxial microstrain model (100 as the unique axis) was used to model the peak profiles. The axial and equatorial microstrains for Co are 7.4 × 10^4^ and 5.6 × 10^4^ ppm, while those for Ni show a greater difference, at 1.1 × 10^5^ and 1.5 × 10^4^ ppm, respectively. This possibly indicates that the Ni compound also contains some alternate metal-ion coordinations (different orientations of the carboxyl groups). During some refinements of the Ni compound, the orientation of the carboxyl groups changed considerably, and/or the displacement coefficients became very large. The very broad peaks of the Ni powder pattern certainly limit the structural information that can be obtained.

The Bravais–Friedel–Donnay–Harker (Bravais, 1866 ▸, Friedel, 1907 ▸; Donnay & Harker, 1937 ▸) morphology suggests that we might expect a platy (with {100} as the major faces) morphology for the compounds. No preferred orientation correction model was necessary in the Co and Ni refinements.

## 3. Supra­molecular features

**Figure 8:**
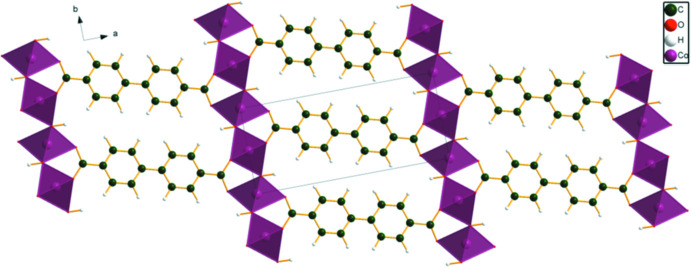
Crystal structure of Co_2_(BPDC)(OH)_2_, viewed down the *c*-axis. Image generated using *DIAMOND* (Crystal Impact, 2022 ▸).

The Mn and Co compounds are isostructural (Fig. 8 ▸). Both *M*14 and *M*15 exhibit an octa­hedral coordination, and occupy centers of symmetry. For *M*14, the coordination consists of *trans* carboxyl­ate O12 atoms and four equatorial hydroxyl groups. For *M*15 there are *trans* hydroxyl groups and four equatorial carboxyl­ate O13 atoms. The bond-valence sums are 1.94 and 2.09 for Mn and 1.80 and 1.85 for Co, in acceptable agreement with the expected values of 2.00. The carboxyl­ate O12 atom bonds to one *M*14, and O13 bridges two *M*15. The hydroxyl group O16 bridges two *M*14 and one *M*15.

The *M*14 octa­hedra share edges to form chains running parallel to the *c*-axis. The *M*15 octa­hedra also share edges to form chains parallel to the *c*-axis. These chains share corners (the O16 OH groups), linking into layers lying parallel to the *bc* plane. The hydroxyl groups do not participate in hydrogen bonds.

**Figure 9:**
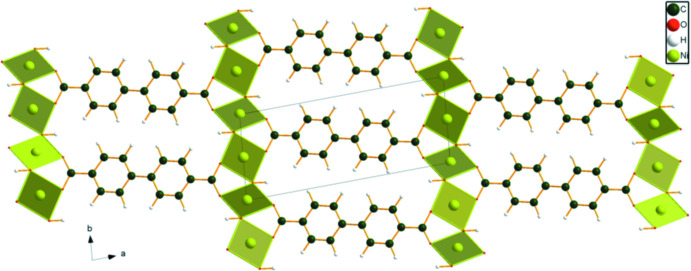
Crystal structure of Ni_2_(BPDC)(OH)_2_, viewed down the *c*-axis. Image generated using *DIAMOND* (Crystal Impact, 2022 ▸).

**Figure 10:**
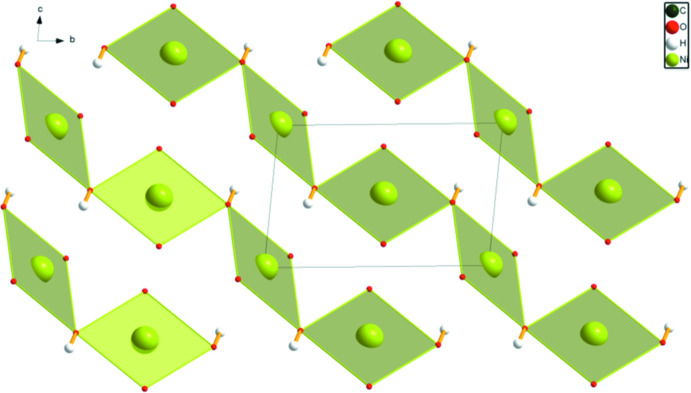
View of the discontinuous layers in Ni_2_(BPDC)(OH)_2_ down the *a-*axis. Image generated using *DIAMOND* (Crystal Impact, 2022 ▸).

The coordination in the Ni compound is different from the other two (Fig. 9 ▸). Ni14 is square planar, with *trans* carboxyl­ate O12 atoms and two *trans* hydroxyl groups. Ni15 is also square planar, with *trans* hydroxyl O16 and carboxyl­ate O13 atoms. Atom O12 is bonded to Ni14 (same), and O13 is bonded to Ni15 (different). Each carboxyl group bridges two metal atoms (not three), and the hydroxyl group O16 bridges one Ni14 and one Ni15. Both Ni ions share hydroxyl corners to form chains lying parallel to the [01 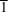 ] axis. The result is layers, but not connected (Fig. 10 ▸).

**Figure 11:**
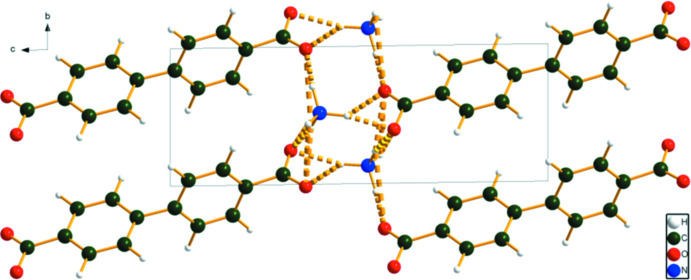
Crystal structure of (NH_4_)_2_(BPDC), viewed down the *a*-axis. Image generated using *DIAMOND* (Crystal Impact, 2022 ▸). The hydrogen bonds are illustrated by heavy dashed lines.

The structure of (NH_4_)_2_BPDC consists of alternating layers of BPDC dianions and ammonium cations lying parallel to the *ab* plane (Fig. 11 ▸). As expected, each hydrogen atom of the ammonium ions in (NH_4_)_2_BPDC participates in a strong N—H⋯O hydrogen bond (Table 1 ▸). The energies of these hydrogen bonds were calculated using the correlation of Wheatley & Kaduk (2019 ▸).

## 4. Database survey

We attempted to solve the structure of Co_2_BPDC(OH)_2_ from the powder data without success. Previous searches of the Cambridge Structural Database [CSD version 5.43 June 2022 (Groom *et al.*, 2016 ▸); *ConQuest* 2022.2.0 (Bruno *et al.*, 2002 ▸)] did not yield suitable analogues, but searches of CSD release 2021.3 using a BPDC fragment and the chemistry CHO and Ni, Zn, Fe, Mn, or Mg only yielded a few hits, among which was Mn_2_BPDC(OH)_2_, refcode UBUPEQ (Sibille *et al.*, 2021 ▸). This compound has a similar powder pattern to our Co and Ni compounds (Fig. 3 ▸), and provided a suitable starting model for Rietveld refinements.

## 5. Synthesis and crystallization

Cobalt(II) nitrate hexa­hydrate (0.4383 g, 1.5 mmol) and biphenyl-4,4′-di­carb­oxy­lic acid (0.3645 g, 1.5 mmol) were added to a flask with 1.5 ml of tri­ethyl­amine and ∼60 ml of di­methyl­formamide (DMF). The mixture was stirred on a hot plate (343 K) until the solution appeared to be homogenous (∼15 min). A 5 ml aliquot of this solution was transferred to a Pyrex microwave vial and heated using a CEM Discover microwave with power set to 150 W using a ramp time of 2 min to reach 423 K with a hold time of 30 min and inter­nal stirring off. Automatic cooling was turned off and the vial was left in the microwave until it cooled to 343 K. The solution was filtered using vacuum filtration and washed with DMF (10 ml). The remaining purple solid was dried in a vacuum oven at ∼343 K.

Nickel(II) acetate tetra­hydrate (0.0880 g, 0.35 mmol) and di­ammonium biphenyl-4,4′-di­carboxyl­ate (0.1278 g, 0.5 mmol) were added to a flask and ∼20 ml of DMF was added. The reaction was stirred on a hot plate (343 K) until solution appeared to be homogenous (∼15 min). A 5 ml aliquot of this solution was transferred to a Pyrex microwave vial and heated using a CEM Discover microwave with power set to 200 W using a ramp time of 5 min to reach 423 K with a hold time of 30 min and inter­nal stirring on high. Automatic cooling was turned on. The solution was filtered using vacuum filtration and washed with DMF (10 ml). The remaining green solid was dried in a vacuum oven at ∼343 K.

0.8990 g (4.1 mmol) of biphenyl-4,4′-di­carb­oxy­lic acid (Aldrich Lot #BCCF5104) were placed into a 50 ml beaker. About 50 ml of 15 *M* aqueous ammonia were placed in a 250 ml beaker, and the 50 ml beaker placed in the larger beaker. The large beaker was covered with a Petri dish, and allowed to stand at ambient conditions overnight. The white recovered solid weighed 1.0257 g, corresponding to the expected qu­anti­tative yield for (NH_4_)_2_BPDC.

## 6. Refinement

Crystal data, data collection and structure refinement details are summarized in Table 2 ▸.

The powder pattern of (NH_4_)_2_BPDC was indexed using *DOCVOL14* (Louër & Boultif, 2014 ▸). All attempts to solve and refine the structure in space group *P* 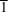 were unsuccessful, so *P*1 was used. The structure was solved by Monte Carlo simulated-annealing techniques as implemented in *EXPO2014* (Altomare *et al.*, 2013 ▸), using a BPDC anion and two N atoms as fragments.

**Figure 12:**
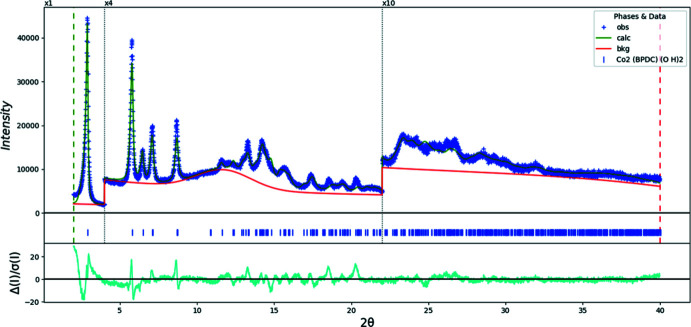
The Rietveld plot for the refinement of Co_2_BPDC(OH)_2_. The blue crosses represent the observed data points, and the green line is the calculated pattern. The cyan curve is the normalized error plot. The row of tick marks indicates the calculated reflection positions. The vertical scale has been multiplied by a factor of 4× for 2θ > 4.0°, and by a factor of 10× for 2θ > 22.0°.

Rietveld refinements (Figs. 12 ▸–14 ▸ ▸) were carried out using *GSAS-II* (Toby & Von Dreele, 2013 ▸). All non-H bond distances and angles in the BPDC dianion were subjected to restraints, based on a *Mercury* Mogul Geometry Check (Sykes *et al.*, 2011 ▸; Bruno *et al.*, 2004 ▸). The Mogul average and standard deviation for each qu­antity were used as the restraint parameters. The restraints contributed 0–2.3% to the final χ^2^. The *U*_iso_ parameters were grouped by chemical similarity: given the complex, low-symmetry structures and poor data quality, these values should be treated with caution. The *U*_iso_ for the H atoms were fixed at 1.3 × *U*_iso_ of the heavy atoms to which they are attached. The peak profiles were described using the generalized microstrain model and the backgrounds were modeled using a 3–12-term shifted Chebyshev polynomial. For Co, the value of μ·*R* used was 0.37. For the ammonium salt, no absorption correction was necessary. For Ni, the geometry was reflection, so no absorption correction was appropriate.

The structures were optimized with density functional techniques using *VASP* (Kresse & Furthmüller, 1996 ▸) (fixed experimental unit cells) through the *MedeA* graphical inter­face (Materials Design, 2016 ▸). The calculations were carried out on 16 2.4 GHz processors (each with 4 Gb RAM) of a 64-processor HP Proliant DL580 Generation 7 Linux cluster at North Central College. The calculations for Co and Ni were spin-polarized magnetic calculations, using the simplified LDSA+U approach, and *U*_J_ = 3.7 for Mn, Co and Ni. The calculations used the GGA-PBE functional, a plane wave cutoff energy of 400.0 eV, and a *k*-point spacing of 0.5 Å^−1^ leading to a 1 × 3 × 4 mesh.

## Supplementary Material

Crystal structure: contains datablock(s) Co_X, Ni_X, UBUPEQ_DFT, NH4_X, global, Co_DFT, Ni_DFT, NH4_DFT. DOI: 10.1107/S2056989022009288/hb8029sup1.cif

CCDC references: 2208530, 2208529, 2208528, 2208527, 2208526, 2208525, 2208524

Additional supporting information:  crystallographic information; 3D view; checkCIF report

## Figures and Tables

**Figure 5:**
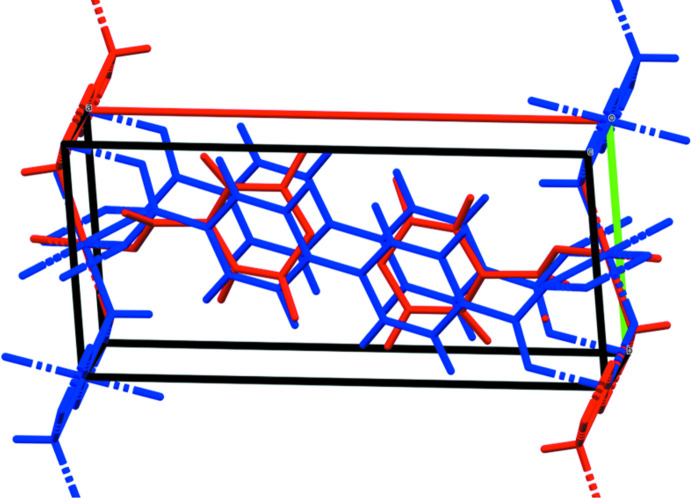
Comparison of the Rietveld-refined (red) and *VASP*-optimized (blue) structures of Co_2_(BPDC)(OH)_2_. The r.m.s. Cartesian displacement is 0.264 Å. Image generated using *Mercury* (Macrae *et al.*, 2020 ▸).

**Figure 6:**
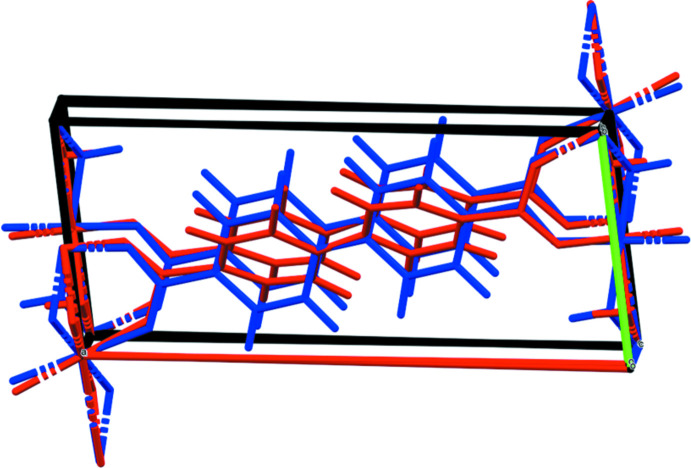
Comparison of the Rietveld-refined (red) and *VASP*-optimized (blue) structures of Ni_2_(BPDC)(OH)_2_. The r.m.s. Cartesian displacement is 0.563 Å. Image generated using *Mercury* (Macrae *et al.*, 2020 ▸).

**Figure 13:**
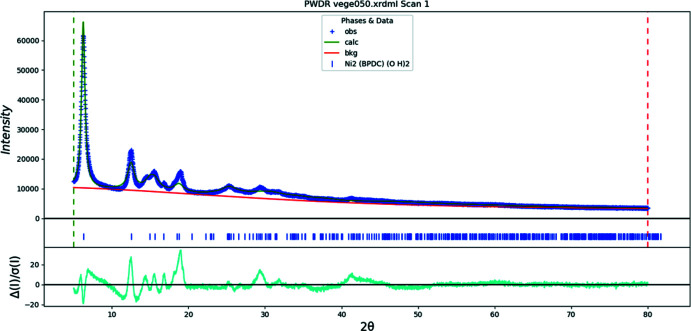
The Rietveld plot for the refinement of Ni_2_BPDC(OH)_2_. The blue crosses represent the observed data points, and the green line is the calculated pattern. The cyan curve is the normalized error plot. The row of tick marks indicates the calculated reflection positions.

**Figure 14:**
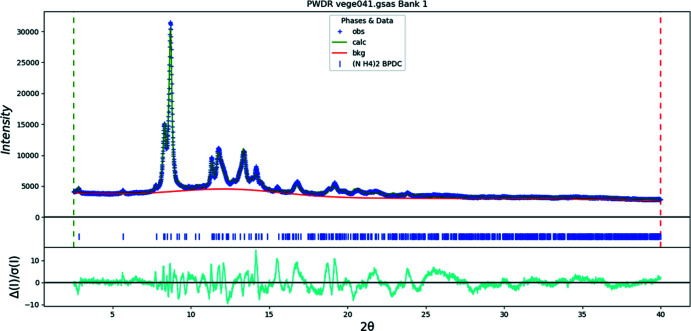
The Rietveld plot for the refinement of (NH_4_)_2_BPDC(OH)_2_. The blue crosses represent the observed data points, and the green line is the calculated pattern. The cyan curve is the normalized error plot. The row of tick marks indicates the calculated reflection positions.

**Table 1:** Hydrogen-bond geometry (Å, °)

*D*—H⋯*A*	*D*—H	H⋯*A*	*D*⋯*A*	*D*—H⋯*A*
N27—H29⋯O25^i^	1.05	1.88	2.907	167
N27—H30⋯O26^ii^	1.04	1.95	2.979	172
N27—H31⋯O24^iii^	1.06	1.62	2.650	162
N27—H32⋯O26^iv^	1.04	1.90	2.942	174
N28—H33⋯O23^v^	1.06	1.62	2.655	164
N28—H34⋯O26^ii^	1.04	2.00	3.007	164
N28—H35⋯O25^i^	1.04	1.88	2.904	169
N28—H36⋯O25	1.05	1.85	2.885	172

Symmetry codes: (i) 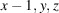 ; (ii) 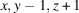 ; (iii) 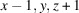 ; (iv) 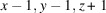 ; (v) 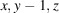 .

**Table 2:** Experimental details

	Co_2_(O_2_CC_6_H_4_C_6_H_4_CO_2_)(OH)_2_	Ni_2_(O_2_CC_6_H_4_C_6_H_4_CO_2_)(OH)_2_	(NH_4_)_2_BPDC
Crystal data			
Chemical formula	[Co(C_14_H_8_O_4_)_0.5_(OH)]	[Ni(C_14_H_8_O_4_)_0.5_(OH)]	2NH_4_^+^·C_14_H_8_O_4_^2−^
*M*_r_	392.09	391.63	276.29
Crystal system, space group	Triclinic, *P* 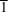	Triclinic, *P* 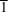	Triclinic, *P*1
Temperature (K)	300	300	300
*a*, *b*, *c* (Å)	14.16 (5), 6.269 (3), 3.323 (4)	15.0 (11), 6.04 (12), 4.04 (9)	4.6770 (6), 5.2306 (14), 14.387 (6)
α, β, γ (°)	91.43 (2), 98.46 (7), 90.0 (3)	82.7 (2), 72.3 (8), 82 (2)	90.57 (7), 91.41 (4), 92.775 (11)
*V* (Å^3^)	291.6 (2)	345 (2)	351.43 (17)
*Z*	1	1	1
Radiation type	*K*α_1,2_, λ = 0.70932, 0.71361 Å	*K*α_1,2_, λ = 1.54059, 1.54445 Å	*K*α_1,2_, λ = 0.70932, 0.71361 Å
Specimen shape, size (mm)	Cylinder, 12 × 0.7	Flat sheet, 16 × 16	Cylinder, 12 × 0.7

Data collection			
Diffractometer	PANalytical Empyrean	PANalytical X’Pert	PANalytical Empyrean
Specimen mounting	Glass capillary	Si zero-background cell with well	Glass capillary
Data collection mode	Transmission	Reflection	Transmission
Scan method	Step	Step	Step
2θ values (°)	2θ_min_ = 1.002 2θ_max_ = 49.991, 2θ_step_ = 0.008	2θ_min_ = 4.008 2θ_max_ = 99.998, 2θ_step_ = 0.017	2θ_min_ = 1.008 2θ_max_ = 49.982, 2θ_step_ = 0.008

Refinement			
*R* factors and goodness of fit	*R*_p_ = 0.065, *R*_wp_ = 0.092, *R*_exp_ = 0.022, *R*(*F* ^2^) = 0.11340, χ^2^ = 21.977	*R*_p_ = 0.042, *R*_wp_ = 0.059, *R*_exp_ = 0.011, *R*(*F* ^2^) = 0.09176, χ^2^ = 30.426	*R*_p_ = 0.033, *R*_wp_ = 0.043, *R*_exp_ = 0.015, *R*(*F* ^2^) = 0.09394, χ^2^ = 14.055
No. of parameters	49	47	93
No. of restraints	64	30	55
(Δ/σ)_max_	2.587	4.433	0.723

The same symmetry and lattice parameters were used for the DFT calculations as for each powder diffraction study. Computer program: *GSAS-II* (Toby & Von Dreele, 2013 ▸).

## supplementary crystallographic information

### Poly[(µ4-4,4'-biphenyldicarboxylato)di-µ-hydroxido-dicobalt] (Co_X) Crystal data

**Table:**

[Co(C_14_H_8_O_4_)_0.5_(OH)]	β = 98.46 (7)°
*M**_r_* = 392.09	γ = 90.0 (3)°
Triclinic, *P*1	*V* = 291.6 (2) Å^3^
Hall symbol: -P 1	*Z* = 1
*a* = 14.16 (5) Å	*D*_x_ = 2.233 Mg m^−^^3^
*b* = 6.269 (3) Å	*K*α_1,2_ radiation, λ = 0.70932, 0.71361 Å
*c* = 3.323 (4) Å	*T* = 300 K
α = 91.43 (2)°	cylinder, 12 × 0.7 mm

### Poly[(µ4-4,4'-biphenyldicarboxylato)di-µ-hydroxido-dicobalt] (Co_X) Data collection

**Table:**

PANalytical Empyrean diffractometer	Scan method: step
Specimen mounting: glass capillary	2θ_min_ = 1.002°, 2θ_max_ = 49.991°, 2θ_step_ = 0.008°
Data collection mode: transmission	

### Poly[(µ4-4,4'-biphenyldicarboxylato)di-µ-hydroxido-dicobalt] (Co_X) Refinement

**Table:**

Least-squares matrix: full	Profile function: Finger-Cox-Jephcoat function parameters U, V, W, X, Y, SH/L: peak variance(Gauss) = Utan(Th)^2^+Vtan(Th)+W: peak HW(Lorentz) = X/cos(Th)+Ytan(Th); SH/L = S/L+H/L U, V, W in (centideg)^2^, X & Y in centideg 30.816, 10.768, 0.000, 1.935, 0.000, 0.033,
*R*_p_ = 0.065	49 parameters
*R*_wp_ = 0.092	H-atom parameters not defined?
*R*_exp_ = 0.022	(Δ/σ)_max_ = 2.587
*R*(*F*^2^) = 0.11340	Background function: Background function: "chebyschev-1" function with 4 terms: 1205(8), -655(9), 147(7), -88(6), Background peak parameters: pos, int, sig, gam: 11.72(4), 4.94(12)e5, 3.12(13)e4, 0.100,
5864 data points	Preferred orientation correction: March-Dollase correction coef. = 1.000 axis = [0, 0, 1]

### Poly[(µ4-4,4'-biphenyldicarboxylato)di-µ-hydroxido-dicobalt] (Co_X) Fractional atomic coordinates and isotropic or equivalent isotropic displacement parameters (Å^2^)

**Table:**

	*x*	*y*	*z*	*U*_iso_*/*U*_eq_	
C1	0.613 (3)	0.625 (7)	0.90 (3)	0.26 (3)*	
C3	0.704 (3)	0.560 (8)	0.84 (3)	0.26 (3)*	
C5	0.7409 (16)	0.364 (3)	0.97 (2)	0.26 (3)*	
C6	0.688 (3)	0.233 (7)	1.18 (3)	0.26 (3)*	
C8	0.594 (3)	0.287 (11)	1.23 (2)	0.26 (3)*	
C10	0.5516 (10)	0.485 (9)	1.077 (11)	0.26 (3)*	
C11	0.8397 (9)	0.301 (2)	0.895 (13)	0.026 (13)*	
O12	0.8581 (8)	0.108 (3)	0.890 (6)	0.026 (13)*	
O13	0.9031 (7)	0.446 (2)	0.938 (3)	0.026 (13)*	
H2	0.58666	0.79555	0.81088	0.3419*	
H4	0.75003	0.66882	0.67401	0.3419*	
H7	0.72054	0.07982	1.31427	0.3419*	
H9	0.54982	0.17404	1.38976	0.3419*	
O16	0.9611 (9)	0.8112 (12)	0.471 (3)	0.0500*	
H17	0.89031	0.81057	0.42272	0.0650*	
Co14	1.00000	0.00000	1.00000	0.018 (3)*	
Co15	1.00000	0.50000	0.50000	0.018 (3)*	

### Poly[(µ4-4,4'-biphenyldicarboxylato)di-µ-hydroxido-dicobalt] (Co_X) Geometric parameters (Å, º)

**Table:**

C1—C3	1.399 (18)	O12—C11	1.232 (10)
C1—C10	1.427 (15)	O12—Co14	2.105 (9)
C3—C1	1.399 (18)	O13—C11	1.272 (11)
C3—C5	1.389 (7)	O16—H17	0.992 (13)
C5—C3	1.389 (7)	O16—Co14^ii^	2.098 (8)
C5—C6	1.385 (8)	O16—Co14^iii^	2.121 (8)
C5—C11	1.503 (8)	O16—Co15	2.028 (8)
C6—C5	1.385 (8)	H17—O16	0.992 (13)
C6—C8	1.41 (3)	Co14—O12	2.105 (9)
C8—C6	1.41 (3)	Co14—O12^iv^	2.105 (9)
C8—C10	1.447 (15)	Co14—O16^v^	2.121 (8)
C10—C1	1.427 (15)	Co14—O16^vi^	2.098 (8)
C10—C8	1.447 (15)	Co14—O16^vii^	2.098 (8)
C10—C10^i^	1.489 (5)	Co14—O16^viii^	2.121 (8)
C11—C5	1.503 (8)	Co15—O16	2.028 (8)
C11—O12	1.232 (10)	Co15—O16^viii^	2.028 (8)
C11—O13	1.272 (11)		
			
C3—C1—C10	121.0 (6)	C1—C10—C8	116.0 (9)
C1—C3—C5	121.4 (5)	C1—C10—C10^i^	114 (5)
C3—C5—C6	119.5 (5)	C8—C10—C10^i^	124 (6)
C3—C5—C11	119.9 (5)	C5—C11—O12	117.3 (8)
C6—C5—C11	120.5 (6)	C5—C11—O13	117.1 (8)
C5—C6—C8	120.5 (9)	O12—C11—O13	123.6 (10)
C6—C8—C10	120.9 (10)		

Symmetry codes: (i) −*x*+1, −*y*+1, −*z*+2; (ii) *x*, *y*+1, *z*; (iii) *x*, *y*+1, *z*−1; (iv) −*x*+2, −*y*, −*z*+2; (v) *x*, *y*−1, *z*+1; (vi) *x*, *y*−1, *z*; (vii) −*x*+2, −*y*+1, −*z*+2; (viii) −*x*+2, −*y*+1, −*z*+1.

### (Co_DFT) Crystal data

**Table:**

C_14_H_10_Co_2_O_6_	α = 91.80°
*M**_r_* = 392.09	β = 99.44°
Triclinic, *P*1	γ = 89.98°
*a* = 14.20000 Å	*V* = 302.23 Å^3^
*b* = 6.23720 Å	*Z* = 1
*c* = 3.46100 Å	

### (Co_DFT) Data collection

**Table:**

*h* = →	*l* = →
*k* = →	

### (Co_DFT) Fractional atomic coordinates and isotropic or equivalent isotropic displacement parameters (Å^2^)

**Table:**

	*x*	*y*	*z*	*B*_iso_*/*B*_eq_	
C1	0.61569	0.62016	0.89476		
C3	0.70973	0.56535	0.88026		
C5	0.74057	0.35498	0.94939		
C6	0.67601	0.20338	1.04253		
C8	0.58279	0.26043	1.06503		
C10	0.54978	0.47000	0.98891		
C11	0.84012	0.29062	0.92724		
O12	0.85873	0.09206	0.91494		
O13	0.90299	0.44077	0.92824		
H2	0.59361	0.78413	0.83081		
H4	0.76000	0.68483	0.81104		
H7	0.70119	0.04101	1.10392		
H9	0.53543	0.13971	1.15004		
O16	0.95981	−0.19591	0.47462		
H17	0.89031	−0.18943	0.42272		
Co14	1.00000	0.00000	1.00000		
Co15	1.00000	0.50000	0.50000		

### Poly[(µ4-4,4'-biphenyldicarboxylato)di-µ-hydroxido-dinickel] (Ni_X) Crystal data

**Table:**

[Ni(C_14_H_8_O_4_)_0.5_(OH)]	β = 72.3 (8)°
*M**_r_* = 391.63	γ = 82 (2)°
Triclinic, *P*1	*V* = 345 (2) Å^3^
Hall symbol: -P 1	*Z* = 1
*a* = 15.0 (11) Å	*D*_x_ = 1.883 Mg m^−^^3^
*b* = 6.04 (12) Å	*K*α_1,2_ radiation, λ = 1.54059, 1.54445 Å
*c* = 4.04 (9) Å	*T* = 300 K
α = 82.7 (2)°	flat_sheet, 16 × 16 mm

### Poly[(µ4-4,4'-biphenyldicarboxylato)di-µ-hydroxido-dinickel] (Ni_X) Data collection

**Table:**

PANalytical X'Pert diffractometer	Scan method: step
Specimen mounting: Si zero-background cell with well	2θ_min_ = 4.008°, 2θ_max_ = 99.998°, 2θ_step_ = 0.017°
Data collection mode: reflection	

### Poly[(µ4-4,4'-biphenyldicarboxylato)di-µ-hydroxido-dinickel] (Ni_X) Refinement

**Table:**

Least-squares matrix: full	47 parameters
*R*_p_ = 0.042	30 restraints
*R*_wp_ = 0.059	H-atom parameters not defined?
*R*_exp_ = 0.011	(Δ/σ)_max_ = 4.433
*R*(*F*^2^) = 0.09176	Background function: Background function: "chebyschev-1" function with 6 terms: 6.12(5)e3, -3.68(4)e3, 8.6(4)e2, 83(31), -134(21), 50(21),
5745 data points	Preferred orientation correction: March-Dollase correction coef. = 1.000 axis = [0, 0, 1]
Profile function: Finger-Cox-Jephcoat function parameters U, V, W, X, Y, SH/L: peak variance(Gauss) = Utan(Th)^2^+Vtan(Th)+W: peak HW(Lorentz) = X/cos(Th)+Ytan(Th); SH/L = S/L+H/L U, V, W in (centideg)^2^, X & Y in centideg 5.186, -8.449, 5.755, 3.463, 0.000, 0.021,	

### Poly[(µ4-4,4'-biphenyldicarboxylato)di-µ-hydroxido-dinickel] (Ni_X) Fractional atomic coordinates and isotropic or equivalent isotropic displacement parameters (Å^2^)

**Table:**

	*x*	*y*	*z*	*U*_iso_*/*U*_eq_	
C1	0.620 (3)	0.56 (4)	−0.23 (3)	0.02 (4)*	
C3	0.707 (2)	0.51 (2)	−0.21 (3)	0.02 (4)*	
C5	0.7272 (18)	0.38 (2)	0.07 (2)	0.02 (4)*	
C6	0.653 (4)	0.30 (3)	0.34 (3)	0.02 (4)*	
C8	0.568 (2)	0.322 (18)	0.32 (2)	0.02 (4)*	
C10	0.546 (3)	0.46 (3)	0.02 (4)	0.02 (4)*	
C11	0.8370 (16)	0.350 (15)	0.074 (19)	0.2200*	
O12	0.873 (4)	0.147 (19)	0.13 (5)	0.2200*	
O13	0.897 (4)	0.50 (2)	−0.066 (13)	0.2200*	
H2	0.60369	0.68368	−0.44499	0.0500*	
H4	0.76708	0.58056	−0.43809	0.0500*	
H7	0.66673	0.210111	0.58822	0.0500*	
H9	0.51034	0.233993	0.52487	0.0500*	
Ni14	1.00000	0.00000	0.00000	0.018 (14)*	
O16	1.00 (2)	−0.179 (4)	0.43 (2)	0.1000*	
H17	0.93829	−0.17184	0.50234	0.1300*	
Ni15	1.00000	0.50000	−0.50000	0.018 (14)*	

### Poly[(µ4-4,4'-biphenyldicarboxylato)di-µ-hydroxido-dinickel] (Ni_X) Geometric parameters (Å, º)

**Table:**

C1—C3	1.31 (2)	C11—O13	1.311 (16)
C1—C10	1.39 (3)	O12—C11	1.297 (10)
C3—C1	1.31 (2)	O12—Ni14	1.942 (14)
C3—C5	1.396 (9)	O13—C11	1.311 (16)
C5—C3	1.396 (9)	O13—Ni15	1.953 (12)
C5—C6	1.404 (16)	Ni14—O12	1.942 (14)
C5—C11	1.642 (12)	Ni14—O12^ii^	1.942 (14)
C6—C5	1.404 (16)	Ni14—O16	1.927 (19)
C6—C8	1.31 (2)	Ni14—O16^ii^	1.927 (19)
C8—C6	1.31 (2)	O16—Ni14	1.927 (19)
C8—C10	1.46 (2)	O16—Ni15^ii^	1.919 (13)
C10—C1	1.39 (3)	Ni15—O13	1.953 (12)
C10—C8	1.46 (2)	Ni15—O13^iii^	1.953 (12)
C10—C10^i^	1.464 (8)	Ni15—O16^iv^	1.919 (13)
C11—C5	1.642 (12)	Ni15—O16^ii^	1.919 (13)
C11—O12	1.297 (10)		
			
C3—C1—C10	122 (2)	C1—C10—C8	117.5 (18)
C1—C3—C5	120.8 (6)	C1—C10—C10^i^	114 (5)
C3—C5—C6	119.0 (9)	C8—C10—C10^i^	128 (8)
C5—C6—C8	120.9 (19)	O12—C11—O13	115.7 (12)
C6—C8—C10	119.7 (8)		

Symmetry codes: (i) −*x*+1, −*y*+1, −*z*; (ii) −*x*+2, −*y*, −*z*; (iii) −*x*+2, −*y*+1, −*z*−1; (iv) *x*, *y*+1, *z*−1.

### (Ni_DFT) Crystal data

**Table:**

C_14_H_10_Ni_2_O_6_	α = 81.57°
*M**_r_* = 391.63	β = 71.90°
Triclinic, *P*1	γ = 81.90°
*a* = 15.10000 Å	*V* = 343.52 Å^3^
*b* = 6.05000 Å	*Z* = 1
*c* = 4.02000 Å	

### (Ni_DFT) Data collection

**Table:**

*h* = →	*l* = →
*k* = →	

### (Ni_DFT) Fractional atomic coordinates and isotropic or equivalent isotropic displacement parameters (Å^2^)

**Table:**

	*x*	*y*	*z*	*B*_iso_*/*B*_eq_	
C1	0.61147	0.63610	−0.07671		
C3	0.70313	0.58310	−0.06941		
C5	0.73667	0.36292	0.03127		
C6	0.67329	0.19956	0.14026		
C8	0.58130	0.25374	0.13082		
C10	0.54811	0.47197	0.01398		
C11	0.83907	0.31116	−0.01437		
O12	0.87153	0.11194	0.07915		
O13	0.88625	0.47867	−0.15724		
H2	0.58988	0.80914	−0.16146		
H4	0.75109	0.71275	−0.14925		
H7	0.69710	0.02821	0.22559		
H9	0.53478	0.12063	0.21394		
Ni14	1.00000	0.00000	0.00000		
O16	0.96416	−0.18943	0.44560		
H17	0.89673	−0.16684	0.55127		
Ni15	1.00000	0.50000	−0.50000		

### (UBUPEQ_DFT) Crystal data

**Table:**

C_14_H_10_Mn_2_O_6_	α = 90.09°
Triclinic, *P*1	β = 96.84°
*a* = 14.20370 Å	γ = 91.71°
*b* = 6.47851 Å	*V* = 315.35 Å^3^
*c* = 3.45320 Å	*Z* = 2

### (UBUPEQ_DFT) Data collection

**Table:**

*h* = →	*l* = →
*k* = →	

### (UBUPEQ_DFT) Fractional atomic coordinates and isotropic or equivalent isotropic displacement parameters (Å^2^)

**Table:**

	*x*	*y*	*z*	*B*_iso_*/*B*_eq_	
C1	0.62098	0.61370	0.92029		
H1	0.60389	0.77429	0.86234		
C2	0.71402	0.55707	0.91323		
H2	0.76792	0.67253	0.85491		
C3	0.73912	0.35104	0.97430		
C4	0.66914	0.20599	0.05646		
H3	0.68946	0.04707	0.11422		
C5	0.57681	0.26460	0.07216		
H4	0.52506	0.14840	0.14642		
C6	0.54945	0.46945	0.99724		
C7	0.83666	0.28496	0.94908		
O1	0.85281	0.09402	0.92904		
O2	0.90327	0.42816	0.95195		
Mn1	1.00000	0.00000	0.00000		
Mn2	1.00000	0.50000	−0.50000		
O3	0.95566	−0.19618	0.47885		
H17	0.886619	−0.19037	0.44315		

### Diammonium 4,4'-biphenyldicarboxylate (NH4_X) Crystal data

**Table:**

2NH_4_^+^·C_14_H_8_O_4_^2^^−^	β = 91.41 (4)°
*M**_r_* = 276.29	γ = 92.775 (11)°
Triclinic, *P*1	*V* = 351.43 (17) Å^3^
Hall symbol: P 1	*Z* = 1
*a* = 4.6770 (6) Å	*D*_x_ = 1.306 Mg m^−^^3^
*b* = 5.2306 (14) Å	*K*α_1,2_ radiation, λ = 0.70932, 0.71361 Å
*c* = 14.387 (6) Å	*T* = 300 K
α = 90.57 (7)°	cylinder, 12 × 0.7 mm

### Diammonium 4,4'-biphenyldicarboxylate (NH4_X) Data collection

**Table:**

PANalytical Empyrean diffractometer	Scan method: step
Specimen mounting: glass capillary	2θ_min_ = 1.008°, 2θ_max_ = 49.982°, 2θ_step_ = 0.008°
Data collection mode: transmission	

### Diammonium 4,4'-biphenyldicarboxylate (NH4_X) Refinement

**Table:**

Least-squares matrix: full	93 parameters
*R*_p_ = 0.033	55 restraints
*R*_wp_ = 0.043	H-atom parameters not defined?
*R*_exp_ = 0.015	(Δ/σ)_max_ = 0.723
*R*(*F*^2^) = 0.09394	Background function: Background function: "chebyschev-1" function with 4 terms: 3149(17), -491(16), 99(12), -147(15), Background peak parameters: pos, int, sig, gam: 12.38(8), 1.18(6)e6, 1.20(8)e5, 0.100,
5862 data points	Preferred orientation correction: Simple spherical harmonic correction Order = 4 Coefficients: 0:0:C(2,-2) = 0.79(3); 0:0:C(2,-1) = 0.32(7); 0:0:C(2,0) = 0.330(31); 0:0:C(2,1) = 1.58(9); 0:0:C(2,2) = 0.88(4); 0:0:C(4,-4) = 0.33(7); 0:0:C(4,-3) = 1.02(5); 0:0:C(4,-2) = 0.65(6); 0:0:C(4,-1) = -0.39(8); 0:0:C(4,0) = -0.79(4); 0:0:C(4,1) = -0.01(9); 0:0:C(4,2) = 1.10(6); 0:0:C(4,3) = 0.79(8); 0:0:C(4,4) = -0.31(7)
Profile function: Finger-Cox-Jephcoat function parameters U, V, W, X, Y, SH/L: peak variance(Gauss) = Utan(Th)^2^+Vtan(Th)+W: peak HW(Lorentz) = X/cos(Th)+Ytan(Th); SH/L = S/L+H/L U, V, W in (centideg)^2^, X & Y in centideg 30.816, 10.768, 0.000, 1.935, 0.000, 0.033,	

### Diammonium 4,4'-biphenyldicarboxylate (NH4_X) Fractional atomic coordinates and isotropic or equivalent isotropic displacement parameters (Å^2^)

**Table:**

	*x*	*y*	*z*	*U*_iso_*/*U*_eq_	
H1	0.56271	0.55748	−0.04903	0.0500*	
C2	0.72650	0.71007	−0.07277	0.0042*	
C3	1.092 (10)	1.091 (7)	−0.1369 (14)	0.0042*	
C4	0.886 (5)	0.858 (4)	−0.0073 (7)	0.0042*	
C5	0.759 (8)	0.741 (7)	−0.1644 (4)	0.0042*	
C6	0.942 (5)	0.928 (5)	−0.1987 (9)	0.0042*	
C7	1.068 (8)	1.055 (6)	−0.0421 (13)	0.0042*	
H8	0.63274	0.60998	−0.21606	0.0500*	
H9	1.19666	1.18586	0.00901	0.0500*	
H10	1.23536	1.25494	−0.16391	0.0500*	
C11	0.935 (5)	0.754 (5)	0.0884 (8)	0.0042*	
C12	1.040 (7)	0.578 (6)	0.2730 (11)	0.0042*	
C13	0.788 (12)	0.847 (10)	0.1638 (15)	0.0042*	
C14	1.140 (8)	0.571 (9)	0.1072 (12)	0.0042*	
C15	1.197 (11)	0.490 (10)	0.1986 (15)	0.0042*	
C16	0.833 (10)	0.754 (9)	0.2538 (12)	0.0042*	
H17	0.62741	1.00179	0.15295	0.0500*	
H18	1.26305	0.48583	0.04787	0.0500*	
H19	1.37364	0.35094	0.21156	0.0500*	
H20	0.69528	0.82586	0.31194	0.0500*	
C21	1.076 (7)	0.480 (7)	0.3737 (13)	0.0566*	
C22	0.944 (5)	0.962 (6)	−0.3033 (10)	0.0566*	
O23	0.840 (7)	0.413 (8)	0.4081 (17)	0.0566*	
O24	0.969 (10)	0.758 (7)	−0.3508 (14)	0.0566*	
O25	1.309 (7)	0.371 (8)	0.4015 (16)	0.0566*	
O26	0.762 (7)	1.119 (7)	−0.3310 (18)	0.0566*	
N27	0.34475	1.28836	−0.39168	0.0500*	
H29	0.36001	1.43931	−0.43695	0.0500*	
H30	0.22878	1.13756	−0.42606	0.0500*	
H31	0.54682	1.23237	−0.37405	0.0500*	
H32	0.24338	1.34422	−0.33265	0.0500*	
N28	0.88714	0.85588	−0.47716	0.0500*	
H33	0.85037	0.66062	−0.48279	0.0500*	
H34	0.69609	0.94431	−0.48449	0.0500*	
H35	1.02320	0.91738	−0.52839	0.0500*	
H36	0.97893	0.90122	−0.41298	0.0500*	

### Diammonium 4,4'-biphenyldicarboxylate (NH4_X) Geometric parameters (Å, º)

**Table:**

C2—C4	1.392 (7)	C16—C13	1.402 (7)
C2—C5	1.342 (6)	C21—C12	1.550 (7)
C3—C6	1.379 (6)	C21—O23	1.258 (9)
C3—C7	1.385 (6)	C21—O25	1.310 (9)
C4—C2	1.392 (7)	C22—C6	1.518 (7)
C4—C7	1.408 (7)	C22—O24	1.269 (9)
C4—C11	1.501 (8)	C22—O26	1.272 (9)
C5—C2	1.342 (6)	O23—C21	1.258 (9)
C5—C6	1.373 (6)	O24—C22	1.269 (9)
C6—C3	1.379 (6)	O25—C21	1.310 (9)
C6—C5	1.373 (6)	O26—C22	1.272 (9)
C6—C22	1.518 (7)	N27—H29	1.0294
C7—C3	1.385 (6)	N27—H30	1.0294
C7—C4	1.408 (7)	N27—H31	1.0295
C11—C4	1.501 (8)	N27—H32	1.0294
C11—C13	1.394 (8)	H29—N27	1.0294
C11—C14	1.410 (7)	H30—N27	1.0294
C12—C15	1.401 (6)	H31—N27	1.0295
C12—C16	1.390 (6)	H32—N27	1.0294
C12—C21	1.550 (7)	N28—H33	1.0295
C13—C11	1.394 (8)	N28—H34	1.0295
C13—C16	1.402 (7)	N28—H35	1.0294
C14—C11	1.410 (7)	N28—H36	1.0293
C14—C15	1.408 (6)	H33—N28	1.0295
C15—C12	1.401 (6)	H34—N28	1.0295
C15—C14	1.408 (6)	H35—N28	1.0294
C16—C12	1.390 (6)	H36—N28	1.0293
			
C4—C2—C5	121.9 (4)	C12—C16—C13	121.6 (3)
C6—C3—C7	119.9 (3)	C12—C21—O23	111.9 (7)
C2—C4—C7	116.5 (4)	C12—C21—O25	121.5 (7)
C2—C4—C11	119.4 (6)	O23—C21—O25	119.6 (8)
C7—C4—C11	120.9 (6)	C6—C22—O24	115.6 (7)
C2—C5—C6	121.7 (3)	C6—C22—O26	112.0 (7)
C3—C6—C5	118.8 (4)	O24—C22—O26	118.2 (8)
C3—C6—C22	123.5 (5)	H29—N27—H30	109.483
C5—C6—C22	117.4 (5)	H29—N27—H31	109.476
C3—C7—C4	121.0 (4)	H30—N27—H31	109.464
C4—C11—C13	120.6 (5)	H29—N27—H32	109.459
C4—C11—C14	122.2 (5)	H30—N27—H32	109.468
C13—C11—C14	117.1 (4)	H31—N27—H32	109.477
C15—C12—C16	117.7 (3)	H33—N28—H34	109.48
C15—C12—C21	123.1 (4)	H33—N28—H35	109.471
C16—C12—C21	119.1 (4)	H34—N28—H35	109.47
C11—C13—C16	121.4 (6)	H33—N28—H36	109.475
C11—C14—C15	121.2 (4)	H34—N28—H36	109.469
C12—C15—C14	120.8 (4)	H35—N28—H36	109.461

### (NH4_DFT) Crystal data

**Table:**

C_14_H_16_N_2_O_4_	α = 90.7300°
*M**_r_* = 276.29	β = 91.3790°
Triclinic, *P*1	γ = 92.7400°
*a* = 4.6875 Å	*V* = 352.86 Å^3^
*b* = 5.2421 Å	*Z* = 1
*c* = 14.3820 Å	

### (NH4_DFT) Data collection

**Table:**

*h* = →	*l* = →
*k* = →	

### (NH4_DFT) Fractional atomic coordinates and isotropic or equivalent isotropic displacement parameters (Å^2^)

**Table:**

	*x*	*y*	*z*	*U*_iso_*/*U*_eq_	
H1	0.59450	0.55016	−0.04709	0.0500*	
C2	0.72650	0.71007	−0.07277	0.0042*	
C3	1.06402	1.11446	−0.14004	0.0042*	
C4	0.92175	0.84164	−0.01188	0.0042*	
C5	0.69564	0.78221	−0.16536	0.0042*	
C6	0.86113	0.98774	−0.19952	0.0042*	
C7	1.09298	1.04187	−0.04764	0.0042*	
H8	0.54136	0.67676	−0.21098	0.0500*	
H9	1.25407	1.13999	−0.00242	0.0500*	
H10	1.20239	1.26916	−0.16643	0.0500*	
C11	0.93949	0.77658	0.08868	0.0042*	
C12	0.94951	0.64759	0.27910	0.0042*	
C13	0.78111	0.91048	0.15313	0.0042*	
C14	1.10777	0.58142	0.12166	0.0042*	
C15	1.11387	0.51792	0.21543	0.0042*	
C16	0.78458	0.84572	0.24662	0.0042*	
H17	0.65053	1.06440	0.12910	0.0500*	
H18	1.23568	0.47852	0.07278	0.0500*	
H19	1.24343	0.36444	0.24034	0.0500*	
H20	0.65294	0.94638	0.29501	0.0500*	
C21	0.93857	0.56900	0.37898	0.0566*	
C22	0.83362	1.07341	−0.29872	0.0566*	
O23	0.76266	0.66899	0.43252	0.0566*	
O24	0.99747	0.98318	−0.35852	0.0566*	
O25	1.09802	0.39029	0.40741	0.0566*	
O26	0.65872	1.24648	−0.31870	0.0566*	
N27	0.16243	0.51693	0.60441	0.0400*	
H29	0.16090	0.49359	0.53198	0.0500*	
H30	0.33993	0.43870	0.63488	0.0500*	
H31	0.13695	0.71068	0.62373	0.0500*	
H32	−0.01991	0.41734	0.62701	0.0500*	
N28	0.59171	0.12978	0.47646	0.0400*	
H33	0.62253	−0.06146	0.45540	0.0500*	
H34	0.58472	0.14431	0.54856	0.0500*	
H35	0.41516	0.20817	0.44485	0.0500*	
H36	0.77650	0.23333	0.45705	0.0500*	

### (NH4_DFT) Hydrogen-bond geometry (Å, º)

**Table:**

*D*—H···*A*	*D*—H	H···*A*	*D*···*A*	*D*—H···*A*
N27—H29···O25^i^	1.05	1.88	2.907	167
N27—H30···O26^ii^	1.04	1.95	2.979	172
N27—H31···O24^iii^	1.06	1.62	2.650	162
N27—H32···O26^iv^	1.04	1.90	2.942	174
N28—H33···O23^v^	1.06	1.62	2.655	164
N28—H34···O26^ii^	1.04	2.00	3.007	164
N28—H35···O25^i^	1.04	1.88	2.904	169
N28—H36···O25	1.05	1.85	2.885	172

Symmetry codes: (i) *x*−1, *y*, *z*; (ii) *x*, *y*−1, *z*+1; (iii) *x*−1, *y*, *z*+1; (iv) *x*−1, *y*−1, *z*+1; (v) *x*, *y*−1, *z*.
